# S100A4 in Cancer Metastasis: Wnt Signaling-Driven Interventions for Metastasis Restriction

**DOI:** 10.3390/cancers8060059

**Published:** 2016-06-20

**Authors:** Mathias Dahlmann, Dennis Kobelt, Wolfgang Walther, Giridhar Mudduluru, Ulrike Stein

**Affiliations:** 1 Experimental and Clinical Research Center, Charité University Medicine, Berlin and Max-Delbrück-Center for Molecular Medicine, Robert-Rössle-Str. 10, 13125 Berlin, Germany; dahlmann@mdc-berlin.de (M.D.); dennis.kobelt@mdc-berlin.de (D.K.); wowalt@mdc-berlin.de (W.W.); giridhar.mudduluru@mdc-berlin.de (G.M.); 2 German Cancer Consortium, Im Neuenheimer Feld 280, 69120 Heidelberg, Germany

**Keywords:** Wnt signaling, colorectal cancer, metastasis, S100A4, intervention

## Abstract

The aberrant activity of Wnt signaling is an early step in the transformation of normal intestinal cells to malignant tissue, leading to more aggressive tumors, and eventually metastases. In colorectal cancer (CRC), metastasis accounts for about 90% of patient deaths, representing the most lethal event during the course of the disease and is directly linked to patient survival, critically limiting successful therapy. This review focuses on our studies of the metastasis-inducing gene S100A4, which we identified as transcriptional target of β-catenin. S100A4 increased migration and invasion *in vitro* and metastasis in mice. In patient CRC samples, high S100A4 levels predict metastasis and reduced patient survival. Our results link pathways important for tumor progression and metastasis: the Wnt signaling pathway and S100A4, which regulates motility and invasiveness. S100A4 suppression by interdicting Wnt signaling has potential for therapeutic intervention. As proof of principle, we applied S100A4 shRNA systemically and prevented metastasis in mice. Furthermore, we identified small molecule inhibitors from high-throughput screens of pharmacologically active compounds employing an S100A4 promoter-driven reporter. Best hits act, as least in part, via intervening in the Wnt pathway and restricted metastasis in mouse models. We currently translate our findings on restricting S100A4-driven metastasis into clinical practice. The repositioned FDA-approved drug niclosamide, targeting Wnt signaling, is being tested in a prospective phase II clinical trial for treatment of CRC patients. Our assay for circulating S100A4 transcripts in patient blood is used to monitor treatment success.

## 1. Wnt Signaling in Colorectal Cancer

In colorectal cancer (CRC), metastasis accounts for about 90% of patient deaths, representing the most lethal event during the course of the disease. Metastasis is directly linked to patient survival, and critically limits successful therapy [1,2]. The development of CRC is a sequential process from normal intestine to adenomatous tissue, adenoma, and finally carcinoma, due to the accumulation of many molecular changes, like gene mutations, loss of epigenetic control, altered gene expression, and constitutive activation of cancer related signaling cascades. Important examples that trigger CRC are adenomatous polyposis coli (APC), Kirsten rat sarcoma viral oncogene homolog (KRAS), β-catenin, SMAD family member (SMAD) 2/4, metastasis associated in colon cancer (MACC) 1, programmed cell death (PDCD) 4, tumor suppressor protein p53, and the unbalanced Wingless-type MMTV integration site family (Wnt) signaling pathway [1,3,4,5,6,7].

**Figure 1:**
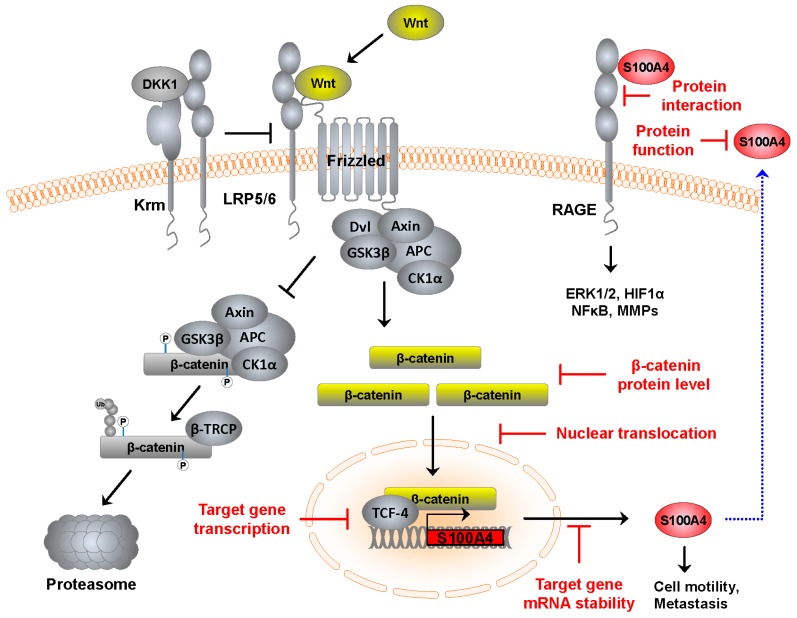
Schematic representation of the Wnt signaling pathway and possible points for therapeutic interventions to restrict S100A4-mediated tumor progression and metastasis. Possible therapeutic intervention points downstream of active Wnt signaling complexes act via reducing β-catenin levels, lowering its nuclear accumulation, and/or inhibiting the formation of active target gene (here S100A4) transcription complexes by small molecules. The expression of specific target genes, such as S100A4, can be reduced by targeting its mRNA level by RNAi. Inhibition of intracellular S100A4 protein function is possible via small molecules. Inhibition of extracellular S100A4 can be achieved with S100A4-specific antibodies, interactions with its receptor (here RAGE) by using receptor-specific antibodies.

Wnt-mediated signaling is one of the crucial signaling pathways in CRC. The molecular composition of the Wnt pathway and its role in signal modulation has been reviewed extensively in the last few years [7,8,9,10], and will also be described here in brief (see Figure 1). In humans 19 evolutionary conserved Wnt genes exist [11]. The Wnt proteins bind to various receptors and activate receptor specific downstream signaling. Mainly, Wnt-mediated pathway activity can be defined as either canonical (*i.e.*, β-catenin dependent) or non-canonical (*i.e.*, β-catenin independent) signaling, subgrouped in non-canonical planar cell polarity (PCP) and non-canonical Wnt/calcium pathway. However, cross-talk downstream of both pathway classes is reported, based on the availability of extracellular Wnts, cellular context, and the types of Wnt receptor [11].

Without active signaling, β-catenin has a rather short half-life in the cytoplasm, since it gets phosphorylated by the so called “destruction complex”, consisting of glycogen synthase kinase (GSK) 3β, casein kinase (CK) Iα, Axin, and APC, subsequent ubiquitinylation and eventual degradation by the proteasome [9].

The canonical Wnt pathway is triggered by the interaction of Wnt with the Frizzled receptor and the co-receptors low-density lipoprotein receptor-related proteins (LRP) 5/6. This inactivates the β-catenin destruction complex, when Axin is recruited by the segment polarity protein dishevelled homolog (Dvl) to the activated Wnt/Frizzled/LRP complex. β-catenin starts to accumulate in the cytoplasm and eventually translocates into the nucleus, where it activates the transcription of target genes under the control of a T-cell factor (TCF) binding motif along with other factors [12].

The activation of the Wnt signaling pathway is tightly controlled at the cell surface. Secreted Wnt-antagonists of the Dickkopf (DKK) family form a ternary complex by binding to the DKK receptor family Kremen (Krm) and the Wnt co-receptor LRP5/6, thereby inhibiting the formation of active Wnt/Frizzled/LRP signaling complexes [13]. The tight control of Wnt/β-catenin signaling is disrupted by an aberrant overexpression of Wnt ligands, mutations in interacting motifs of destruction complex molecules, or mutations in β-catenin itself [9,14,15]. The key regulator of the destruction complex, APC, is mutated in around 80% of the CRC tumor specimens [16]. The destruction complex is also inactivated, when frameshift mutations occur in Axin2 [17]. Besides this, 15% of the remaining CRC tumor specimens harbor mutations in β-catenin [18]. The most important mutations in β-catenin occur at the amino acid S45 in exon 3, which is phosphorylated by CK1α and at S33, S37, T41, which get phosphorylated by GSK3β in the active destruction complex. When phosphorylation at these sites is not possible, either by loss of the destruction complex or mutation of crucial phosphorylation sites, β-catenin will not be triggered for proteasomal degradation and will induce aberrant Wnt signaling target gene transcription [1,19].

## 2. S100A4

The S100 calcium binding protein A4 was first discovered by Ebradlize and colleagues in 1989 and was initially named metastasin (mts1) [20]. Already in this initial report, the metastatic potential of S100A4 was unveiled. Later, it was independently cloned by several groups and several names were given, such as fibroblast-specific protein (FSP1), 18A2, pEL98, p9Ka, 42A, CAPL, and calvasculin (reviewed in [21]).

### 2.1. S100—Family and Function

S100A4 belongs to a family of S100 proteins, named due to their solubility in saturated ammonium sulfate. The first member of the group of S100 proteins was described back in 1965 by Moore [22]. Currently, more than 20 proteins that belong to this gene family are known. Most of them can be found at human chromosome 1 (1q21), where they form two clusters, containing S100A1-9 and S100A12-16 at one locus, and S100A10 and 11 at another position [23]. The remaining coding sequences for S100 proteins can be found on different chromosomes throughout the genome, including the X chromosome. S100 proteins are a highly similar group of small Ca^2+^ binding proteins with a molecular mass of 10–12 kDa, which share 50% of their amino acid sequence. This high degree of homology suggests a common ancestor, that might have evolved 500 million years ago [24]. All S100 proteins share an EF hand motif as common structural feature. The S100 proteins act as homo- or heterodimers, or can form oligomers, as exemplified by S100A4. Each monomer contains two EF hand motifs, that in most cases are Ca^2+^ binding helix-loop-helix domains [25]. The N-terminally located domain (pseudo EF hand) is composed of 14 amino acids while the C-terminal canonical EF hand is built of 12 amino acid residues. The latter binds Ca^2+^ with higher affinity [26]. Binding of Ca^2+^ to the EF hand motif results in a movement of the two helices giving access to hydrophobic protein-protein interaction sites, which were previously hidden in the protein structure. The Ca^2+^ bound S100 proteins are regarded as ”open” and constitute the active form [27,28]. S100 proteins have been shown to be involved in numerous different cellular functions, e.g., proliferation, differentiation, apoptosis, calcium homeostasis, metabolism, inflammation and motility (reviewed in [29]). All S100 family members have no known enzymatic activity and exert their role via interaction with and regulation of other proteins. Depending on the given S100 protein, they can act intracellularly, in the extracellular space, or in both compartments [30].

Most of the S100 family members are involved in or initiating biological functions contributing to malignant disease such as proliferation, metastasis, angiogenesis and immune evasion. These proteins represent promising candidates for cancer diagnosis and prognosis as well as therapeutic targets with first inhibitors already identified and tested in clinical trials (reviewed in [31]).

### 2.2. S100A4—a β-Catenin Target Gene

Since Wnt signaling activity is altered in almost all CRC tumors, we addressed the question, which genes are modified in their expression by a gain-of-function (GOF) mutation in β-catenin [32]. We performed a gene expression profile analysis with the human CRC cell line HCT116 (heterozygous of wt and Δ45-mutant β-catenin, lacking serine 45) and HAB-92^wt^ cells, a HCT116 derived cell line expressing monoallelic wt β-catenin [32]. In this array we observed 40-fold increase of S100A4 expression in HCT116 cells, compared to HAB-92^wt^ cells. This result was confirmed in HCT116-derived cell lines HAB-68^mut^, expressing monoallelic Δ45-mutant β-catenin, and NCI-H28^null^, nullosomic for β-catenin, verifying, that functionally active β-catenin is necessary for S100A4 induced expression in CRC. Forced overexpression or knock-down of S100A4 and/or mutated β-catenin further proved, that transcriptionally active β-catenin enhances the S100A4-induced migration and invasion of HCT116, HAB-92^wt^ and NCI-H28^null^ cells. In a CRC xenograft mouse model, intrasplenic or intracardiac transplantation of HAB-92^wt^ cells, with or without stable mutant β-catenin expression, demonstrated that due to GOF in the mutant β-catenin, this group showed more metastatic lesions in liver and lungs, compared to vector control cells. With this evidence we have designed a comprehensive study to determine whether S100A4 is a direct transcriptional target of β-catenin. Sequence analysis of the human S100A4 promoter revealed a putative TCF binding motif at −679 to −673. Luciferase experiments with or without TCF binding site mutations showed that S100A4 is a direct transcriptional target of β-catenin. Further, the binding of β-catenin to the S100A4 promoter was confirmed by electrophoretic mobility shift assays (EMSA) and chromatin immunoprecipitation (ChIP) analysis [32]. This data suggests that S100A4 is indeed a direct transcriptional target of the Wnt/β-catenin/TCF-mediated signaling pathway, strongly suggesting novel therapeutic interventions or screening for pharmacologically active compounds to reduce S100A4 expression in CRC.

### 2.3. S100A4 in Non-Malignant Disease

S100A4 is associated with both non-malignant and malignant human diseases. Several groups have reported a role of S100A4 in inflammation. Human articular chondrocytes upregulate S100A4 expression levels during rheumatoid- and osteoarthritis. Elevated S100A4 levels lead to increased phosphorylation of protein tyrosine kinase (Pyk)-2, mitogen-activated protein (MAP) kinases, and activated nuclear factor kappa-light-chain-enhancer of activated B cells (NFκB), that in turn results in elevated matrix metalloproteinase (MMP)-13 secretion. These effects are mediated at least in part via the receptor for advanced glycation end products (RAGE), as the inhibition of RAGE resulted in decreased S100A4 dependent signaling [33]. When analyzing bone material from patients with osteoarthritis, by employing microarrays and quantitative PCR, the authors show an upregulation of S100A4, but also other Wnt-related genes [34]. S100A4 was also shown to be commonly overexpressed in cardiac hypertrophy [35]. The expression of S100A4 in this model tissue injury was linked to general elevated expression of cell growth related proteins, leading to tissue remodeling during reconstitution of the myocardium. Here S100A4 acts as growth factor and pro-survival factor in the myocard [36]. Later, the role of S100A4 during cardiomyogenesis was described *in vitro* [37].

### 2.4. S100A4 in Cancer

The cellular functions of S100A4 were mainly characterized in cancer, promoting tumor progression and metastasis formation, reviewed by Boye and Mælandsmo, and recently by Bresnick and colleagues [31,38]. Enhanced cell growth and motility upon elevated S100A4 expression increases the metastatic potential of cancer cells originating from many entities, like breast, lung, prostate, bone, and cancers from the digestive tract, *in vitro* and in mice [31]. The expression level of S100A4 in tumors of cancer patients also correlates with enhanced progression and metastasis formation, emphasizing its importance in clinical cancer diagnosis. This has been observed for many cancer types, including bladder cancer [39], breast cancer [40], lung squamous cell carcinoma [41], pancreatic carcinoma [42], gastric [43], and colorectal cancer [32]. S100A4 expression in cancer, besides the above mentioned Wnt/β-catenin pathway, is mediated by the receptor tyrosine-protein kinase erbB (ERBB) 2, which upregulates S100A4 via extracellular signal-regulated kinase (ERK) signaling in medulloblastoma [44]. Interestingly, this mechanism might lead to a feed-forward loop in S100A4 expression regulation, since extracellular S100A4 itself was reported to stimulate ERBB2 receptor signaling [45]. S100A4 expression in breast cancer also depends on integrin signaling via the proto-oncogene tyrosine-protein kinase Src and nuclear factor of activated T-cells (NFAT) 5, specifically using integrin α6β4 response to epithelial mesenchymal transition (EMT), promoting cell motility [46,47].

EMT is an important step in cancer development, characterized by aberrant signaling activities, including the Wnt pathway (reviewed in [48]). Before S100A4 was known as a Wnt signaling target gene, its expression was reported to be an early factor involved in the process of EMT in epithelial cells [49]. The role of S100A4 in tumor progression and metastasis via induction of EMT has been confirmed in many types of cancers, including CRC [50,51,52,53]. There, S100A4 and also β-catenin were found higher expressed in the tumor invasive margin [53,54].

When focusing on the molecular mechanisms of S100A4 and its role in cancer, a number of cancer related protein-protein interaction partners have been described, including cytoskeletal proteins such as actin, myosin, and tropomyosin (reviewed in [27]). An important example for S100A4-induced motility is mediated via its interaction with non-muscle myosin-II, where S100A4 can negatively regulate polymerization of myosin-IIA filaments by interacting with the C-terminal part of its heavy chain [55,56]. A higher disassembly rate of myosin-IIA filaments, especially at leading edges of migrating cells, contributes to cell motility and metastasis formation [57]. A very recent aspect of S100A4-dependent mechanisms at the plasma membranes of tumor cells has been published by Jaiswal and colleagues. The authors describe the repair of lesions at the plasma membrane as a critical mechanism for migrating and invading cancer cells, subjected to altered membrane stability and higher mechanical tension. Injuries at the membranes are followed by an influx of extracellular Ca^2+^, which in turn triggers the fusion of non-secretory vesicles to seal the wound and the shedding of the injured part [58]. Interestingly, important factors of the repair mechanism, like Ca^2+^-binding annexin A2, filamentous (F-) actin and myosin II, which were described to establish the wound closure in cooperation with S100A11, were also reported to interact with S100A4 [27,58]. Indeed, accumulated S100A4 has been found at sites of plasma membrane repair, pointing to a role of S100A4 in maintaining the invasive potential of tumor cells [58].

S100A4 is also secreted into the intercellular fluid—by the tumor cell itself or by cells in the local tumor environment—and can exert multiple functions by interaction with receptors like RAGE [59,60,61]. RAGE-mediated signaling by extracellular S100A4 leads to nuclear translocation of intracellular S100A4, linking extracellular protein levels to intracellular responses [62]. Besides this, S100A4-induced but RAGE-independent effects can be shown under RAGE negative conditions, e.g., neurite outgrowth, cell motility, and capillary like growth [63,64,65]. For CRC, our group described the hyperactivity of hypoxia response and ERK signaling, leading to increased cellular motility [66]. But also newer reports link S100A4-mediated RAGE signaling to an increase in metastatic potential in cancer, like thyroid cancer and melanoma [67,68].

Taken together, it is well established that S100A4 has profound impact in many types of solid cancers, where its upregulation causes tumor progression and metastasis formation. S100A4 expression levels in tumors are considered as a biomarker for the prognosis of both metachronous metastasis and survival of cancer patients.

#### 2.4.1. Prognostic Value of S100A4 in CRC Tissue

When looking at CRC prognosis in general, there is a decrease of the 5-year survival rate of CRC patients after resection of the primary tumor from approximately 85%, when tumors were diagnosed at stages I and II, to less than 50%, when lymph node metastases occurred (stage III) [69]. In case of distant metastases at time of diagnosis (stage IV), the 5-year survival rate of CRC patients drops to less than 10% [2]. A meta-analysis of eight studies analyzing the prognostic value of S100A4 expression on overall and disease-free survival in CRC consolidates the correlation of high S100A4 expression in tumor tissues with low survival rates of the patients [70]. We analyzed CRC patients with previously non-metastasized primary tumors with regard to their S100A4 expression in the tumors and their disease prognosis. Immunohistochemical staining of S100A4 in tumor tissue and gene specific quantification of micro-dissected S100A4-mRNA by quantitative reverse-transcription (q-RT) PCR showed higher S100A4 expression in patients, who developed metachronous metastases within 36 months. Overall and metastasis-free survival of patients with S100A4 expressions in the primary tumors above the calculated cut-off were significantly shorter than for patients with low S100A4 expression [32]. A similar analysis was performed in a cohort of 60 CRC patients of stages I-III. Again, S100A4 expression in primary tumors was higher in patients with metastases after tumor resection, and both the overall and metastasis free survival differed significantly, depending on the S100A4 expression [66]. The intracellular and intratumoral distribution of S100A4 proteins is also of prognostic value, as nuclear localization of S100A4 increases the risk for poor survival and metastasis formation in stage II CRC patients [71]. Further, the expression of S100A4 in the advancing tumor front can be used as an independent indicator for overall survival [72].

Today, S100A4 expression in tumor tissue is a valid and valuable biomarker for determining the risk for metastasis formation of CRC patients, and much effort is made to evaluate S100A4 expression in tumors as a predictor for therapy response.

#### 2.4.2. Diagnostic and Prognostic Value of Circulating S100A4 Transcripts in Patient’s Blood

The link of reported prognostic value of high S100A4 levels in the primary tumor for metachronous metastasis and reduced patient survival, documented in a large body of studies, is mainly based on snapshot analyses due to tissue availability. Thus, we established for the first time a non-invasive, plasma-based assay for the quantification of circulating S100A4 transcripts in blood of colon, rectal, and gastric cancer patients, that allows clinical application routinely for diagnosis, prognosis and for monitoring treatment success [73]. We determined increased S100A4 transcripts in cancer patients of each entity and all disease stages, compared with tumor-free volunteers, with sensitivities of 96%, 74%, and 90% and specificities of 59%, 82%, and 71%, for colon, rectal, and gastric cancer patients, respectively. In prospectively analyzed follow-up patients, higher S100A4 levels were found in those patients who later experienced metastasis, compared with patients without metastasis. In high S100A4-expressing patients, disease-free survival was decreased. Thus, we demonstrated the diagnostic and prognostic potential of this plasma-based assay for early defining patients’ risk for metastasis. Currently, we are employing this assay for monitoring circulating S100A4 transcripts levels to assess the treatment responses in a clinical phase II trial.

Combinatorial detection of relevant transcripts might even enhance diagnosis, prognosis, and/or prediction for cancer patients. Thus we combined detection of circulating transcripts of S100A4 with those of the MACC1 gene for CRC and gastric cancer patients [74,75]. We discovered the gene MACC1 in 2009 [76]. It is meanwhile acknowledged as prognostic and predictive biomarker for tumor progression and metastasis linked to patient survival for a broad variety of solid tumor entities [77,78]. S100A4 as Wnt/β-catenin target gene [53,54] as well as MACC1 [79] were independently found at the tumor invasion front in CRC patients. Both genes are considered to be crucially involved in CRC liver metastasis [80]. When combining S100A4 with circulating transcripts of MACC1, improved survival prediction was seen for newly diagnosed CRC as well as gastric cancer patients [74]. Interestingly, a combination with β-catenin levels might also be an option, since increased levels of β-catenin have also been determined in CRC patient plasma correlating with tumor stage [81]. Very recently, Barbazan and colleagues reported the prognostic relevance of a S100A4/MACC1 cluster in circulating tumor cells for progression-free and overall survival of patients with metastatic CRC [82].

## 3. Therapeutic Interventions of S100A4-Mediated Tumor Progression and Metastasis

Elevated S100A4 expression is altering the genetic expression pattern of cells, leading to a more malignant phenotype [38]. For CRC patients, higher tumor stages at time of diagnosis and the risk to develop metastases are drastically shortening their life span. Therefore, there is a need for therapeutic options to either reduce the expression level of S100A4 in the tumor or its environment, or to interfere with downstream effects of the protein, like protein-protein interactions and signaling pathway modulation. Applicable methods to reduce the S100A4-dependent metastatic potential include the knock-down of S100A4-mRNA via RNAi or the therapy with small molecules, screened for interfering in cellular S100A4 functions or S100A4 promoter activity. With the focus on Wnt signaling, we will review drugs, which affect S100A4 expression by inhibiting this very crucial pathway.

### 3.1. RNAi-Based Knock-Down of S100A4 Expression

Early attempts to reduce the mRNA and protein level of S100A4 in the late 1990s reported successful reduction of the S100A4-induced metastatic phenotype for osteosarcoma, *in vitro* as well as *in vivo* [83]. Ribozyme-based knock-down of S100A4 in cultured CRC cells verified the decrease of cellular motility. The reduction in S100A4 protein levels also altered the cellular matrix remodeling genes, like MMPs and tissue inhibitors of metalloproteinases (TIMPs), responsible for the invasion of cancer cells into surrounding tissues. In cell culture, S100A4-specific siRNA reduced the expression MMP-9 and MMP-10, but increased TIMP-4 [84]. Our group also found reduced MMP-9 levels in tumors of CRC xenografted mice, after hydrodynamics-based systemic treatment with plasmids coding for S100A4-specific shRNA, via repeated tail vein injection [85]. This treatment also decreased the formation of liver metastases significantly in those animals, verifying the role of S100A4 in CRC metastasis formation [32]. Additionally to cellular invasion, increased angiogenesis has been found in S100A4-related cancer, when extracellular S100A4 binds to the endothelial plasminogen co-receptor annexin 2 and plasminogen itself [86]. Other reports link extracellular S100A4 to vascular endothelial growth factor (VEGF) expression and metastasis formation [87,88]. But also the RNAi-based knock-down of S100A4 directly in thyroid cancer cells reduced VEGF expression, in addition to MMP-9, and thus invasion and angiogenesis [89]. For CRC, the connection of S100A4 overexpression and elevated VEGF levels, resulting in increased viability and migration, was reported recently [90].

Taken together, the reduction of S100A4 expression, either in the tumor itself or in its environment, has been proven to reduce the metastatic potential of CRC, shown by decreased cell motility *in vivo*, as well as in less metastasis formation *in vivo*. Applying RNAi-based therapeutics to decrease S100A4 expression in the clinic might be an approach to reduce the metastatic burden of CRC patients and may prolong their disease-free survival.

### 3.2. Sulindac

Sulindac is long known as a nonsteroidal anti-inflammatory drug (NSAID), which inhibits the cyclooxygenase (COX) activity of the prostaglandin endoperoxide synthase (PTGS1 and PTGS2) enzyme that is involved in inflammation processes by converting arachidonic acid to prostaglandin H2. However, in the 1970s it came into focus that such drugs, including sulindac, also exert anti-tumor effects, which was repeatedly confirmed by clinical studies [91,92]. The sulindac metabolite sulindac sulfide, which is generated in the liver, is responsible for this anti-tumor and chemopreventive action [93].

#### 3.2.1. Sulindac and Wnt Signaling

More detailed studies on the molecular mechanism of action of sulindac revealed that the anti-tumor and the chemopreventive activity of this compound is rather COX-independent, as shown in different cancer cell lines with varying levels of COX-expression [94]. More interestingly, these anti-tumor effects of sulindac have been linked to its intervention with Wnt signaling, namely β-catenin transcriptional activity in association with reduced nuclear accumulation or reduced non-phosphorylated levels of β-catenin [95,96]. This link was further supported by the chemopreventive activities of sulindac in the rodent azoxymethane carcinogen model, which closely resembles the clinical situation of β-catenin and APC mutant colon cancers [97,98]. In line with this, clinical trials with sulindac have shown significant reduction in colorectal polyps in familial and in sporadic adenomatous polyposis patients [99,100]. In fact these data paved the ground for analyzing the anti-tumor and anti-metastatic efficacy of sulindac in the context of S100A4, as one attractive target of the Wnt signaling pathway [32].

#### 3.2.2. Sulindac as a S100A4 Inhibitor

In our studies we also used the pharmacological inhibitor sulindac known to intervene in the Wnt signaling pathway. We demonstrated the reduction of β-catenin-mediated S100A4 activation and expression in GOF as well as in loss of function (LOF) β-catenin variant carrying human CRC cell lines treated with sulindac [101]. The property of increased cellular migration and invasion in GOF lines was decreased by 30% to 60% with sulindac treatment. The expression knock-down of β-catenin by sulindac led to its reduced nuclear accumulation and to reduced binding of β-catenin to TCF-4. This resulted in decreased S100A4 promoter activity and S100A4 expression. This correlated well with inhibition of cell migration and invasion, which was rescued by ectopic cytomegalovirus (CMV)-promoter driven S100A4 overexpression. Sulindac administration in mice, intrasplenically transplanted with colon cancer cells which were transfected with mutant β-catenin, revealed reduced tumor growth and metastasis formation compared with solvent treated control animals. Sulindac treatment resulted in significantly reduced β-catenin as well as S100A4 mRNA and protein levels in the spleen tumors, compared to solvent treated controls. Also in the liver metastases, β-catenin and S100A4 levels were lowered by sulindac treatment. These *in vitro* and *in vivo* experiments demonstrate that S100A4-mediated tumor progression and metastasis formation, driven by β-catenin signaling, can be mitigated with administration of sulindac. This further exemplifies the effective interference of a NSAID compound in Wnt signaling as anti-tumor and anti-metastatic mode of action. The attractiveness of such an approach is supported by the fact, that meanwhile new sulindac derivatives such as sulindac-benzylamine were developed, which also inhibit colon cancer cell growth by suppression of transcriptional activity of β-catenin [96]. Another derivative, NOSH-sulindac, a nitric oxide- and hydrogen sulfide-releasing hybrid, has been reported recently, which also exerts anti-tumor activities in numerous cancer cell lines [102].

### 3.3. Novel Transcriptional Inhibitors of S100A4

The knowledge on the importance of S100A4 for metastasis formation and its involvement in the Wnt signaling pathway is the driving force for continued search for novel inhibitors. This search is accelerated by the use of high throughput assays, which provide the technological platform to identify promising new drug candidates from drug libraries [103]. Such libraries may contain already known and FDA-approved drugs or encompass large numbers of novel compounds with quite unknown activities.

Regarding the Wnt target S100A4, the key prerequisite for high throughput screening (HTS) was the availability and molecular characterization of the human S100A4 promoter to create the appropriate luciferase-reporter system for the drug screens. The analysis of the S100A4 promoter revealed its link to the Wnt signaling pathway at transcriptional level by the identification of TCF-4 binding sites as functional units of TCF-4/β-catenin-mediated regulation of this gene. For identification of S100A4 transcription inhibitors we performed HTS, using the Library of Pharmacologically Active Compounds (LOPAC) of FDA-approved compounds [104,105]. We employed a CRC cell line stably expressing a human S100A4-promoter driven luciferase reporter gene construct (HCT116/pS100A4-LUC) to screen the library. Those compounds were considered active, which did significantly and specifically reduce S100A4 promoter-driven luciferase expression at preferably low drug concentration and lowest toxicity. The initial HTS, which generated promising hits for active compounds, was followed by validation screens, providing more detailed information on exact values of drug concentration ranges for proper reporter expression inhibition and more precise definition of the respective half maximal effective concentration (EC_50_) values. By this HTS-based workflow two drug candidates were identified, which possessed high inhibitory efficacy of S100A4 expression: the antibiotic calcimycin and the anti-helminthic niclosamide.

#### 3.3.1. Calcimycin

Using the HTS approach with the S100A4-promoter driven reporter assay the polyether antibiotic drug calcimycin was identified with effective inhibitory activity [106,107]. Further analysis of the calcimycin action in HCT116 human CRC cells revealed the transcriptional inhibition of S100A4 expression in a concentration- and time-dependent manner. Similar calcimycin-mediated effects were seen in other human colon cancer cell lines. This observation is paralleled by the previous report that calcimycin is also able to reduce S100A4 expression in mammary adenocarcinoma cells, as well as in monocytes and lymphocytes [108]. This inhibition of S100A4 expression resulted in reduced proliferation, colony formation, and migratory activity of the calcimycin-treated cells. The respective rescue experiments with ectopic CMV-promoter driven overexpression of S100A4 indicated that the calcimycin action is indeed based on the transcriptional inhibition of this gene. The study further revealed the link of calcimycin activity and its potential to intervene in the constitutively active Wnt pathway [109], with other Wnt target genes such as cyclin D1, c-myc, and DKK-1 also being reduced by the drug. Due to the fact that S100A4 is tightly associated with metastasis formation, we also performed *in vivo* studies in mice after intrasplenical application of calcimycin-treated CRC cells to analyze its anti-metastatic activity. These studies showed that calcimycin reduced metastasis formation to 30% compared with control mice. Via bioluminescence imaging and immunohistochemistry, smaller and less frequent liver metastases were seen in the calcimycin treated animals. S100A4 levels in the metastases were also reduced by calcimycin. These *in vivo* studies provide strong indication, that the calcimycin-mediated reduction of S100A4 expression is able to restrict metastasis formation *in vivo* via transcriptional inhibition [106].

#### 3.3.2. Niclosamide

Out of the 1280 well-characterized small molecules of the LOPAC library we identified niclosamide as the most efficient inhibitor of S100A4 promoter activity. Niclosamide is an anti-helminthic drug and is approved for human use since the middle 1960s. It restricts glucose uptake, oxidative phosphorylation and anaerobic metabolism in its target cells [110]. Niclosamide acts as a teniacide and belongs to most essential drugs needed for basic health [111].

##### 3.3.2.1. Niclosamide as Transcriptional S100A4 Inhibitor

Selecting a candidate drug from the HTS is dependent on the ratio of luciferase activity inhibition and cell viability. Niclosamide inhibited luciferase activity at 0.78 μM and higher concentrations, but reduced cell viability at 3.1 μM and higher concentrations. For target gene validation, we analyzed the concentration- and time-dependency of niclosamide to modify endogenous S100A4 mRNA and protein levels in cell culture. To achieve the maximum S100A4 expression inhibition accompanied by minimum toxicity, we went further with a daily application of 1 μM niclosamide resulting in constantly reduced S100A4 expression. Since S100A4 is a major inducer of cell motility, we tested for the effects of niclosamide on S100A4-induced migration and invasion in a panel of human CRC cells. Niclosamide reduced significantly migration, invasion and also impaired wound closure in a wound healing assay in the different CRC cell lines, compared to solvent-treated controls. In rescue experiments using ectopically CMV-promoter driven S100A4 overexpressing CRC cell lines, neither reduction of S100A4 expression nor of cell motility inhibition was seen after niclosamide treatment. Niclosamide also inhibited anchorage-dependent and -independent proliferation of the CRC cells, however, this effect was—in contrast to the inhibition of cell motility—not S100A4-specific. Thus, niclosamide inhibited S100A4-dependent cell motility and invasiveness in CRC cells.

Further, we demonstrated that niclosamide restricted liver metastasis formation by using an intrasplenically xenografted mouse model. Niclosamide was administered intraperitoneally on a daily basis. Tumor growth and metastasis development was monitored by non-invasive *in vivo* luminescence imaging and at the experimental endpoint at day 22 by *ex vivo* imaging of the isolated organs spleen and liver. Whereas solvent-treated control mice formed metastases in the livers, no metastasis development was seen in niclosamide-treated animals. In the spleen tumors of the niclosamide-treated mice, expression of S100A4 was reduced indicating that niclosamide reduced S100A4 levels *in vivo* as well. We also investigated metastasis formation *in vivo* under continuous and discontinuous (until day 24, then solvent-treated until day 50) niclosamide treatment. Both niclosamide-treated mouse groups showed enhanced overall survival compared with the solvent-treated mice linked to long-term inhibition of tumor growth and liver metastasis formation, and to reduction of S100A4 expression. No statistically significant differences were found between the continuously and discontinuously treated animals. We conclude that niclosamide has the potential for the clinical treatment or prevention of CRC metastasis in humans.

##### 3.3.2.2. Niclosamide and Wnt Signaling

A large variety of studies demonstrated actions of niclosamide as anti-cancer agent in different tumor entities, including CRC, breast cancer, prostate cancer, ovarian cancer, non-small cell lung cancer (NSCLC), glioblastoma, osteosarcoma, multiple myeloma, and leukemia. There is growing evidence that anti-cancer actions of niclosamide are predominantly mediated via the Wnt/β-catenin signaling pathway, which is known to represent a major regulatory pathway for cancer initiation, growth, cell differentiation and metastasis [9]. With more than 90% of all the CRC patients harboring mutations in the Wnt/β-catenin signaling pathway, drugs intervening in this pathway came into focus. This anti-cancer drug repositioning is described for the anti-helminthic drug niclosamide for several tumor entities [112].

We also analyzed the effect of niclosamide on the Wnt signaling pathway since we identified the metastasis inducing gene S100A4 as a transcriptional target gene of Wnt/β-catenin signaling [32]. We used HCT116 CRC cells, heterozygous for mutated β-catenin and constitutively active in Wnt signaling and S100A4 expression, as well as isogenic subline derivatives thereof carrying exclusively the mutant or the wildtype β-catenin allele. Treatment with 1 μM niclosamide significantly reduced Wnt pathway activity, S100A4 expression, and consistently, reduced cell migration rates. Since we did not observe altered nuclear levels of β-catenin following niclosamide treatment, we analyzed for formation of the β-catenin/TCF activating complex. We found by EMSA and ChIP that treatment of CRC cells with increasing concentrations of niclosamide interrupted the β-catenin/TCF/oligonucleotide complex in a concentration-dependent manner, leading to its disappearance at concentrations of 1 μM. Taken together, niclosamide treatment inhibited the formation of β-catenin/TCF complex, thereby inhibiting the transcription of the Wnt/β-catenin target gene S100A4.

Also by employing libraries of FDA-approved drugs by HTS, Chen and colleagues identified niclosamide as a drug able to interfere with the Wnt signaling pathway. They demonstrated that niclosamide is able to inhibit Wnt/Frizzled-1 signaling in osteosarcoma cells [113,114]. They showed enhanced Frizzled-1 endocytosis, downregulated Dvl2 protein, thereby inhibiting Wnt3A-stimulated β-catenin stabilization and lymphoid enhancer-binding factor (LEF)/TCF reporter activity. They conclude that niclosamide may serve as a negative modulator of Wnt/Frizzled-1 signaling by depleting upstream signaling molecules. DiRenzo and colleagues showed recently that using niclosamide as Frizzled receptor inhibitor for blocking Wnt signaling abolished transforming growth factor (TGF)-β/SMAD3-induced β-catenin stabilization, influencing smooth muscle cell proliferation [115]. Lu and colleagues analyzed the effect of niclosamide on the essential Wnt co-receptor for Wnt/β-catenin signaling, LRP6 [116]. They showed in HEK293 cells and in human prostate and breast cancer cells, that niclosamide suppressed LRP6 expression and phosphorylation, blocked Wnt3A-induced β-catenin accumulation, and inhibited Wnt/β-catenin signaling, resulting in induced apoptosis and anti-cancer activity with half maximal inhibitory concentration (IC_50_) values less than 1 μM. They concluded that niclosamide has potential as chemopreventive and therapeutic agent for human cancer. Osada and colleagues demonstrated anti-proliferative actions of niclosamide in CRC cells regardless of mutations in APC [117]. This effect was mediated via downregulation of the Wnt signaling pathway. In particular, they showed a decreased expression of Dvl2, leading to reduced downstream β-catenin signaling. In CRC xenografted mice, the authors described a tumor control by orally applied niclosamide treatment and suggest clinical reposition of the drug niclosamide for CRC treatment. Niclosamide effects were also analyzed in hepatoma cells, since Wnt signaling plays also a role in hepatocarcinogenesis [118]. The authors showed reduced cell proliferation following niclosamide treatment, induction of apoptosis, decreased TOP activity, and decreased levels of β-catenin, Dvl2 and cyclin D1. They summarize that niclosamide is a potential candidate for hepatoma treatment. Ono and colleagues reported that niclosamide inhibited proliferation of primary human leiomyoma cells in a dose-dependent manner [119]. Niclosamide-induced proliferation reduction was not related to decreased cell survival due to similar lactate dehydrogenase (LDH) activity levels. The authors showed that niclosamide inhibited the Wnt/β-catenin pathway activation in the human leiomyoma cells by inhibiting the TOP activity and down-regulated pathway target genes like Axin2. Further, they demonstrated reduced nuclear β-catenin translocation after niclosamide treatment. Londoño-Joshi and colleagues demonstrated higher niclosamide-induced cytotoxicity in aldehyde dehydrogenase (ALDH)-enriched non-adherent cells, compared with adherent cells from basal-like breast cancers [120]. Again, niclosamide reduced levels of LRP6 and β-catenin. In combination with TRA-8, an antibody specific to TNF-related apoptosis-inducing ligand (TRAIL) death receptor 5, Wnt signaling was further reduced, *in vitro* cytotoxicity was enhanced, and growth of orthotopic tumor xenografts was suppressed. The authors conclude that niclosamide or its congeners might be beneficial for the treatment of basal-like breast cancers. The effect of niclosamide as a potential therapeutic agent interfering with the Wnt signaling pathway was also investigated for ovarian cancer [121]. The authors treated tumor cells isolated from patients’ ascites with primary ovarian cancer and showed increased cytotoxicity, reduction of Wnt/β-catenin signaling by TOP assay, and decreased Wnt pathway proteins e.g., Axin2 and cyclin D1. The authors evaluate niclosamide as a potent Wnt/β-catenin inhibitor and as a treatment option for ovarian cancer. King and colleagues also investigated niclosamide effects in ovarian cancer models [122]. Niclosamide abrogated Wnt7A/β-catenin signaling, inhibited β-catenin transcriptional activity and cell viability, and decreased cell migration following an increase in E-cadherin and a decrease of SLUG. Niclosamide applied orally inhibited tumor growth and progression in an intraperitoneal xenograft mouse model representative of human ovarian cancer, suggesting niclosamide as a promising inhibitor of this pathway with potential clinical relevance. Recently, Satoh and colleagues identified niclosamide as most promising candidate by HTS for adrenocortical carcinoma [123]. The authors demonstrated that niclosamide inhibited cell proliferation, induced caspase-dependent apoptosis and G1 cell cycle arrest, and decreased cell migration. It also reduced the level of mediators of epithelial-to-mesenchymal transition, decreased expression of β-catenin, and inhibited tumor growth with no observed toxicity in mice.

##### 3.3.2.3. Niclosamide and Further Signaling Pathways

Although all of these studies demonstrate the interference of niclosamide in the Wnt signaling pathway, leading to reduced target gene expression and resulting in reduced cell proliferation and motility in several cancer types, it has to be mentioned that niclosamide is able to block multiple signaling pathways, which play a major role in cancer initiation, progression, and metastasis in a panel of different cancer types. This broad anti-cancer activity is also reflected by a screen with niclosamide on the NCI-60 human tumor cell line panel [124]. Niclosamide was able to inhibit cell proliferation of all lines with IC_50_ values below 1 μM [125]. Several groups investigated the effects of niclosamide to multiple signaling pathways in addition or besides the Wnt signaling pathway. Osada and colleagues identified the Wnt signaling to be targeted by niclosamide and excluded niclosamide-mediated inhibition of NFκB or mTOR pathways in their CRC model systems [117]. Despite intervening in the Wnt signaling pathway it was demonstrated in the last years that niclosamide is able to block different pathways in different cancer types (reviewed for cancer cells and cancer stem cells in [125,126]): niclosamide inhibited the transcription factors E2F1 and AP1, and c-myc-responsive reporters, whereas the hypoxia-inducible factor (HIF) 1α, TCF/LEF, cyclic AMP-responsive element-binding (CREB), NFκB, SMAD/TGF-β, and recombining binding protein suppressor of hairless (RBPJ)/neurogenic locus notch homolog protein (Notch) pathway reporters were inhibited to a lesser extend in human osteosarcoma cells [127]; inhibited the mTOR complex 1 pathway activity by lysosomal dysfunction in different cancer types [128,129,130]; inhibited signal transducer and activator of transcription (STAT) 3 in head and neck cancer cells and in NSCLC cells [131,132], and blocked STAT3 phosphorylation and its translocation into the nucleus in prostate cancer cells [133]; inactivated the NFκB pathway in acute myelogenous leukemia stem cells [134], and simultaneously inhibited Wnt/β-catenin, Notch, mTOR, and NFκB signaling cascades in human glioblastoma [135].

##### 3.3.2.4. Niclosamide and Derivatives

Based on the promising anti-cancer effects of niclosamide, niclosamide chemotypes have been identified or synthesized, and tested for their anti-cancer effects and for their interference with the Wnt signaling pathway. When we discovered the anti-migratory and anti-invasiveness effects of niclosamide via inhibiting the Wnt signaling pathway and the metastasis gene S100A4, we extended our analyses to structural derivatives of this drug [104]. In contrast to the effects of niclosamide itself, we did neither observe reduced levels of S100A4 mRNA and protein levels nor reduced cell migration with any of the six niclosamide derivatives analyzed so far. Thus, changes in the structure of niclosamide described in our study resulted in loss of its efficiency toward inhibition of S100A4 expression and cell motility. Mook and colleagues also found inhibition of Wnt signaling by niclosamide appears unique among the structurally-related anti-helminthic agents they tested [136]. They showed the potency and functional response was dependent on small changes in the chemical structure of niclosamide. The same group published two years later their structure-activity studies of Wnt/β-catenin inhibition by different niclosamide chemotypes [137]. The authors investigated the structure-activity relationships of Wnt signaling inhibition in the anilide and salicylamide region of niclosamide. They identified drug candidates for treating cancers with dysregulated Wnt signaling, including drug-resistant cancers. Walters Haygood and colleagues tested more soluble niclosamide-like analogs on tumorspheres from ovarian cancer patient ascites and slices from solid tumor samples [138]. They found down-regulation of Wnt pathway-associated proteins in the patient samples treated with niclosamide analogs, suggesting these compounds may be useful for the treatment of ovarian cancer. Taken together, these newly developed niclosamide analogs act in cell culture, in mice, as well as in patient samples by intervening in the Wnt/β-catenin signaling pathway [137,138].

##### 3.3.2.5. Niclosamide in Clinical Trials

Based on the niclosamide-related findings reviewed here, we now aim at the repositioning of the anti-helminthic drug niclosamide as anti-cancer agent for the clinical treatment or prevention of colon cancer metastasis. Niclosamide is well tolerated in humans, approved for human use, and 2 g are administered daily in adults to treat tapeworm infections. We are translating our findings on restricting S100A4-driven metastasis into clinical practice. Together with the Charité Comprehensive Cancer Center, we are evaluating the repositioned FDA-approved drug niclosamide targeting Wnt signaling for treatment of CRC patients in a prospective phase II clinical trial: Drug Trial to Investigate the Safety and Efficacy of Niclosamide Tablets in Patients with Metastases of a Colorectal Cancer Progressing after Therapy (Nikolo). This clinical trial is registered with ClinicalTrials.gov (NCT02519582) and the European Clinical Trials Database (EudraCT 2014-005151-20). We initiated this first phase II clinical trial in August 2015 evaluating the safety and efficacy of orally applied niclosamide in patients who are progressive with metachronous or synchronous metastases of CRC after the previous therapy. Two grams niclosamide per day are given to the CRC patients of this monocentric open-label clinical trial, received until progression (according to RECIST = Response Evaluation Criteria in Solid tumor) or unacceptable toxicity. The primary endpoint is the progression-free survival (PFS) at 4 months. Secondary outcome measures are overall survival (date of randomization until date of death, assessed up to 2 years, or date from patient inclusion to date of death or date of last follow-up news, censured data), time to progression (date of randomization until date of first documented progression, assessed up to 2 years; progression according to RECIST criteria), disease control rate (date of randomization, assessed up to 2 years, remission/partial remission/stable disease), the number of adverse events > grade 2 toxicities according to NCI Common Toxicity Criteria for Adverse Effects v4.03 (date of randomization, assessed up to 1 month after end of therapy), and the number of serious adverse events (date of randomization, assessed up to 1 month after end of therapy). The first patients are already enrolled; a total of 37 patients will be enrolled in this interventional trial. The primary outcome measure, PFS at 4 months, will be analyzed in a first evaluation after treatment of the first 17 patients. Paralleling this clinical trial, translational research is performed, e.g., determining S100A4 expression in tumor tissues and metastases, and monitoring treatment success by quantifying the circulating S100A4 transcripts in patient blood. The trial is planned to be completed in February 2018.

Very recently, in February 2016, another clinical trial testing niclosamide in CRC patients was initiated by the Duke University Medical Center: A Study of Niclosamide in Patients with Resectable Colon Cancer. This clinical trial is registered with ClinicalTrials.gov (NCT02687009). The authors are performing this phase I clinical trial to obtain safety data along with pharmacokinetic data and with the determination of the maximum tolerated dose (three dosage levels of niclosamide) in patients with colon cancer who undergo primary resection of their tumor. Further, they wish to obtain information on the changes in the Wnt pathway signaling following niclosamide administration in humans. They aim at future studies in patients with more advanced CRC or other cancers with dysregulation of the Wnt pathway.

Taken together, these recently initiated clinical trials will provide first insights in the niclosamide-induced inhibition of Wnt/β-catenin signaling and the clinical consequences thereof. The outcome of these clinical trials on niclosamide for treatment of CRC patients will contribute to the evaluation of the repositioning of this anti-helminthic drug niclosamide for the clinical treatment or prevention of CRC metastasis.

## 4. Conclusions

Metastasis formation is the major hurdle in CRC therapy. To identify patients at high risk for metastasis formation, early diagnosis and molecular characterization of the primary tumor is crucial to define prognostic and therapeutic targets. S100A4 has been shown to contribute to both demands in CRC: serving as biomarker, which allows the prognosis of patient survival and metachronous metastasis already in early stages being determined in tumor tissue or non-invasively in liquid biopsies; and serving as therapeutic target, since its down-regulation by e.g., small molecules restricts metastasis formation in mice and is currently tested in clinical trials. Other strategies of therapeutic interventions tested in preclinical studies, like S100A4-specific antibodies or drug/peptide-based interference of S100A4-protein interactions, further point to the relevance of treating CRC by targeting S100A4.

As S100A4 is a direct target of β-catenin/TCF-mediated transcription in CRC, interfering in the Wnt signaling pathway thereby reducing expression and/or nuclear accumulation of β-catenin has emerged as new option to restrict S100A4-induced cell motility and metastasis. Promising, already FDA-approved drugs were identified to reduce β-catenin-mediated S100A4 gene transcription resulting in diminished cellular motility *in vitro* and metastasis formation *in vivo* and are currently evaluated in clinical trials to reduce the risk of metastasis formation in CRC thereby improving patient survival and quality of life. In recent years, further approaches have been demonstrated to interfere with the Wnt signaling pathway upstream of β-catenin [139,140]. Their usefulness, however, to restrict S100A4-induced cell motility and metastasis remains to be demonstrated. It might be anticipated that a combinatorial treatment using drugs such as the FDA-approved and repositioned compounds discussed in this review together with inhibitors acting upstream of β-catenin and thus targeting the Wnt signaling pathway at different levels might be most efficient for the patients.

## Abbreviations

The following abbreviations are used in this manuscript:

APC	adenomatous polyposis coli
ChIP	chromatin immunoprecipitation
CK	casein kinase
CMV	cytomegalovirus
COX	cyclooxygenase
CRC	colorectal cancer
DKK	Dickkopf-related protein
Dvl	segment polarity protein dishevelled homolog
EMSA	electrophoretic mobility shift assay
EMT	epithelial-mesenchymal transition
ERBB2	receptor tyrosine-protein kinase erbB-2
ERK	extracellular signal-regulated kinase
FDA	Food and Drug Administration
GOF	gain-of-function
GSK	glycogen synthase kinase
HTS	high throughput screening
IC_50_	half maximal inhibitory concentration
LEF	lymphoid enhancer-binding factor
LOPAC	Library of Pharmacologically Active Compounds
LRP	low-density lipoprotein receptor-related protein
MACC1	metastasis associated in colon cancer 1
MMP	matrix metalloproteinase
mRNA	messenger RNA
NFκB	nuclear factor kappa-light-chain-enhancer of activated B cells
Notch	neurogenic locus notch homolog protein
NSAID	nonsteroidal anti-inflammatory drug
NSCLC	non-small cell lung cancer
PCR	polymerase chain reaction
PFS	progression-free survival
RAGE	receptor for advanced glycation end products
RECIST	response evaluation criteria in solid tumor
RNA	ribonucleic acid
RNAi	RNA interference
S100A4	S100 calcium binding protein A4
si/shRNA	small interfering/hairpin RNA
SMAD	mothers against decapentaplegic homolog
STAT	signal transducer and activator of transcription
TCF	T-cell factor
TGF	transforming growth factor
TIMP	tissue inhibitor of metalloproteinases
VEGF	vascular endothelial growth factor
Wnt	wingless-type MMTV integration site family member
